# Pennsylvania College of Dental Surgery

**Published:** 1882-05

**Authors:** 


### PENNSYLVANIA COLLEGE OF DENTAL SURGERY.

The twenty-sixth annual commencement.

The number of matriculates for the session was one hundred and twenty-five.

The degree of D.D.S. was conferred on the following graduates by Professor S. D. Gross, President of the Board of Trustees:

| NAME. | RESIEENCE. |
|---|---|
| Don A. Allen | Ohio. |
| M. E. Andrews | Pennsylvania. |
| Thos. S. Atkinson | Pennsylvania. |
| C. W. Barber | Pennsylvania. |
| Paul Bayerle | Germany. |
| G. A. Bianchi | Italy. |
| O. C. Bogardus | New Jersey. |
| N. E. Bowman | Pennsylvania. |
| Geo. H. Butler | New York. |
| W. P. Caldwell | Ohio. |
| John Carter | Connecticut. |
| James Granger | New York. |
| J. L. Graves | New York. |
| Charles Harker | New Jersey. |
| E. W. Harris | New York. |
| Alwin Hennet | Germany. |
| D. Rhine Hertz | Pennsylvania. |
| Jasper N. Jones | Florida. |
| Emil Kruger | Germany. |
| L. H. Leitzell | Pennsylvania. |
| J. A. Libbey | Ohio. |
| F. D. Mann | Illinois. |
| F. Mannhardt | Germany. |
| Julius Neumunz | Switzerland. |
| E. Everett Park | Kentucky. |

| NAME. | RESIDENCE. |
|---|---|
| Alfred D. Clark | Pennsylvania. |
| John M. Cooper | Pennsylvania. |
| C. B. Cowan | South Carolina. |
| L. H. De Lange | Pennsylvania. |
| Chas. A. Dougherty | Ohio. |
| J. P. Eldridge | Pennsylvania. |
| M. H. Fetzer | Pennsylvania. |
| O. H. Franklin | Pennsylvania. |
| D. L. German | Pennsylvania. |
| Adolph Gerstel | Germany. |
| Chas. L. Gibbs | Pennsylvania. |
| Harold S. Patterson | Minnesota. |
| D. T. Pepper | Pennsylvania. |
| C. H. Peter | Pennsylvania. |
| H. K. Edward Poessel | Illinois. |
| Chas. J. Rathbun | Pennsylvania. |
| W. L. Reed | Missouri. |
| L. Restrepo | U.S. of Col'bia. |
| A. J. Sawyer | Pennsylvania. |
| Max J. Sternberg | Germany. |
| W. H. Stryker | Pennsylvania. |
| J. C Townsend | Delaware. |
| M. T. Vogle | Pennsylvania. |
| S. P. Waugaman | Pennsylvania. |
| T. R. Whiting | New York. |

